# Technique modifications for septodermoplasty: an illustrative case

**DOI:** 10.1186/s40463-015-0112-4

**Published:** 2015-12-30

**Authors:** Mark Bastianelli, Shaun J. Kilty

**Affiliations:** Department of Otolaryngology-Head and Neck Surgery, University of Ottawa, The Ottawa Hospital, 737 Parkdale Ave., Room 459, Ottawa, ON K1Y 1J8 Canada; Ottawa Hospital Research Institute (OHRI), Ottawa, ON Canada

**Keywords:** Septodermoplasty, Hereditary hemorrhagic telangiectasia, Epistaxis

## Abstract

**Background:**

Hereditary hemorrhagic telangiectasia (HHT) is an autosomal dominant disease that results in telangiectasia of the sinonasal tract, gastro-intestinal tract as well as possible arteriovenous malformations of the lung, liver and brain. One of the most common disease manifestations of HHT is epistaxis. Severe recurrent epistaxis necessitating iron therapy and blood transfusion is often managed with septodermoplasty. Its initial description was as an open surgical technique requiring nasal packing.

**Case presentation:**

We describe a modified approach to septodermoplasty done completely endoscopically and without nasal packing for a patient with severe epistaxis due to HHT.

**Conclusion:**

The described technique modifications for the presented case allowed for same day discharge following surgery, complete take of the skin graft and resultant epistaxis control that ended thepatient's transfusion dependency. The merits of these modifications should be further evaluated in a clinical trial.

## Background

Hereditary hemorrhagic telangiectasia (HHT) is an autosomal dominant disease, which results in blood vessel malformation most commonly within mucous membranes and the skin. In the late 19^th^ century Osler, Weber and Rendu described a series of patients with a constellation of symptoms including frequent epistaxis, recurrent gastrointestinal bleeding, iron deficiency anemia and multiple telangiectasia on the vermillion border of the lips and fingertips [1–3]. These patients were subsequently recognized as a definite medical entity known as Osler Weber Rendu syndrome, now otherwise known as HHT.

The common mucosal vascular malformation that occurs in HHT, telangiectasia, often bleed spontaneously or with minimal trauma. Although the initial clinical disease presentation can vary, the most common symptom of patients with HHT is recurrent epistaxis. Epistaxis is typically the earliest sign of the disease while mucocutaneous and gastrointestinal telangiectasia develop progressively with age [4]. The average age of onset for epistaxis is 12 years of age, with nearly 100 % of patients affected by epistaxis by age 40 [5–7].

Treatment for HHT-related epistaxis is focused on the reduction of the frequency and severity of bleeding. Multiple modalities have reportedly been used including oral tranexamic acid [8], and topical estrogen-containing ointment [9]. A relatively new modality of therapy has been Bevacizumab, a monocolonal antibody inhibitor of vascular endothelial growth factor (VEGF) [10]. Previous studies have demonstrated that intransal administration of Bevacizumab can significantly diminish the frequency and severity of epistaxis in this patient population [11–13]. Although strict medical management can reduce symptom severity, many patients are refractory to pure medical measures and require oral iron supplementation and/or regular blood transfusions. For patients who have failed medical therapy surgical options, including laser coagulation or septodermoplasty, are often employed. Laser ablation however usually requires multiple sessions and is less effective for lesions greater than 2 mm in diameter [14]. Currently, the gold standard for severe recurrent epistaxis for patients with HHT is septodermoplasty. This technique was initially described in 1962 by Saunders et al [15] and it involves the removal of affected nasal epithelium and its replacement with a split-thickness skin graft (STSG). A variation of this technique for severe epistaxis management, known as Young’s procedure [16] involves a septodermoplasty with complete nasal closure.

The objective of this report is to use an illustrative case to describe technique alterations for septodermoplasty that may improve patient care by providing an alternative for improved surgeon visualization, for rapid hemostasis and for secure STSG placement without nasal packing.

## Case presentation

A 64 year old female known for HHT is referred to our clinic for recurrent epistaxis for nearly 50 years. She has had recurrent symptoms since the age of 16 when her condition was diagnosed. All three of her siblings also were diagnosed with HHT and her mother passed away from an intracranial hemorrhage. Eight years prior to presentation she had undergone a left-sided septodermoplasty via a lateral rhinotomy approach. This operation had significantly reduced the frequency of her symptoms and for several years her epistaxis was under control with the use of low dose thalidomide. However, she was referred to our clinic due to increased epistaxis severity and frequency over the prior 12 months necessitating more frequent transfusions.

At the time of consultation the patient was concerned about daily severe left sided epistaxis despite several months use of topical bevacizumab and oral tranexamic acid. She required intravenous iron and blood transfusions every two months. Her baseline hemoglobin at the time of our consultation was 75 g/L (normal = 120 – 160 g/L). Her HHT epistaxis severity score [17] was severe (normalized score 9.49). On examination, she had multiple telangiectasia on her fingers, face, lips and palate. Her endoscopic examination revealed bilateral telangiectasia along the nasal septum. There was extensive crusting along the entire left nasal cavity with which any manipulation resulted in immediate profuse epistaxis. Given the severity of the patient’s epistaxis despite medical therapy, she was offered endoscopic left-sided septodermoplasty. The surgical goals were to improve her quality of life by reducing the number and severity of epistaxis episodes while diminishing the need for blood transfusions. The patient was content with the treatment plan and agreed to undergo surgical intervention.

### Operative procedure

**Fig. 1:**
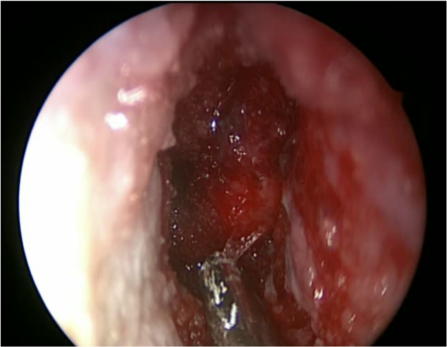
Left septodermoplasty demonstrating application of Floseal for hemostasis

**Fig. 2:**
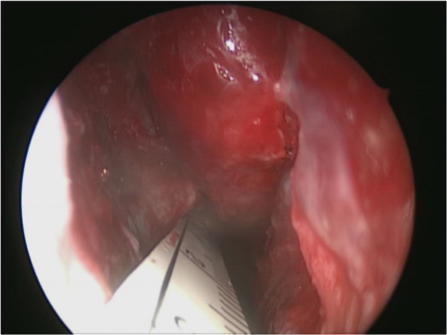
Left septodermoplasty demonstrating the septal mucosal defect

The endoscopic procedure was performed under general anesthesia with endotracheal intubation. The nasal cavity was prepared by inserting pledgets soaked in topical adrenaline (1:1000) placed in both nostrils for decongestion. Using a zero degree endoscope the residual STSG and mucosa of the left septum was dissected in a supraperichondrial plane that resulted in the expected significant diffuse hemorrhage. Immediate hemostasis was attained using a topical gelatin-thrombin matrix, Floseal (FloSeal Hemostatic Matrix; Baxter Healthcare Corporation, Deerfield, IL, USA) (Fig. 1). The mucosal defect (Fig. 2) measured approximately 3 cm in anterior-posterior dimension.

**Fig. 3:**
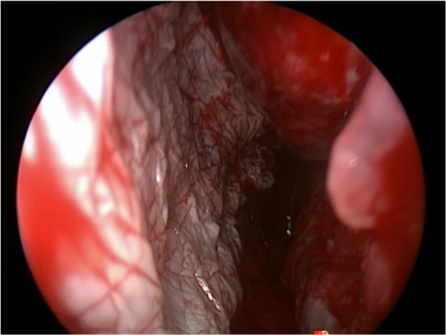
Left septodermoplasty demonstrating endoscopic STSG placement

**Fig. 4:**
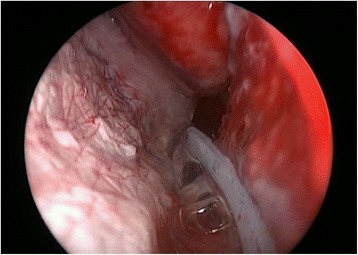
Endoscopic application of TISSEEL sealant over the STSG

A 4 x 2 cm split thickness skin graft was harvested from the right thigh, pie-crusted with a 15 blade and then placed endoscopically along the length of the septal defect. As seen in Fig. 3, the graft was placed with an overlap of the mucosa of the nasal floor and the residual superior septal mucosa. Finally, 2 mL of fibrin sealant (TISSEEL fibrin sealant, Baxter Healthcare Corporation, Deerfield, IL, USA) was then applied first to the edges then central portion of the STSG (Fig. 4). Packing was not used post-operatively and the patient was discharged home on the same day of surgery.

### Post-operative course

**Fig. 5:**
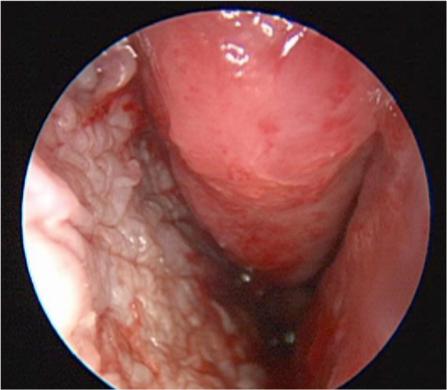
Endoscopic image at two weeks follow-up post left sided septodermoplasty

Clinical follow-up two weeks after surgery (Fig. 5) showed that the entire graft had taken and the left-sided epistaxis had dramatically diminished. The patient was very content with the results of the procedure. At 6 months follow-up, her baseline hemoglobin had improved to 102 g/L and she was requiring transfusions every 4 months with her hematologist’s intent to stop the transfusions if her hemoglobin remained greater than 100 g/L. Her epistaxis severity score at 6 month follow up was mild (normalized score 3.05).

## Discussion

The frequency and duration of epistaxis is a major determinant of quality of life in patients with HHT [18–21]. Septodermoplasty has been shown to adequately decrease symptom severity in 80-100 % of patients up to 6 months post-operatively [22–24]. Many variations of a septodermoplasty have previously been described. Saunders originally described using a lateral rhinotomy approach, which continues to be the most common approach to septodermoplasty. Other variations have included closure of nasal valve (Young’s procedure) [16] as well as buccal mucosal grafts instead of a STSG [25].

Although different approaches have previously been reported we submit that the technique described herein provides several advantages. Firstly, under endoscopic guidance optimal visualization for the entire surgical field is achieved. Ultimately, this allows for increased precision when excising the mucosa as well as ensuring accurate placement of the STSG. Second hemostasis is readily and rapidly achieved with the use of a topical gelatin-thrombin matrix following excision of the nasal septal mucosa. A dry surgical field is imperative when placing the STSG as this diminishes the likelihood of graft hematoma and subsequent failure of the graft [26–28]. Finally, by using a fibrin tissue glue, nasal packing is not required to secure the graft, arrest bleeding, nor to prevent post-operative hematoma. Further, nasal packing can often be a significant source of pain and morbidity in the immediate post-operative period [29, 30]. The colonisation of nasal packing by *Staphylococcus aureus* could also result in STSG loss due to its subsequent infection and disrupted healing [29–32].

## Conclusion

Endoscopic septodermoplasty is a minimally invasive method of performing this classically open surgical procedure. Minimizing blood loss in this chronically anemic patient population is imperative, the use of a topical gelatin-thrombin matrix allows for rapid hemostasis for a sizeable surgical wound. This also provides a dry surgical bed to receive the skin graft and the use of fibrin tissue glue is a packing-free method of securing the skin graft that decreases postoperative patient discomfort as well as decreasing the chances of skin graft loss due to infection from nasal packing bacterial colonisation. We suggest that future studies should compare long-term efficacy of this technique and its cost-benefit with open septodermoplasty.

## Consent

Written informed consent was obtained from the patient for publication of this Case report and any accompanying images. A copy of the written consent is available for review by the Editor-in-Chief of this journal.
